# The growing challenge of antimicrobial resistance in the South-East Asia Region - Are we losing the battle?

**DOI:** 10.4103/0971-5916.73313

**Published:** 2010-11

**Authors:** Rajesh Bhatia, Jai P. Narain

**Affiliations:** Department of Communicable Diseases WHO Regional Office for South-East Asia Mahatma Gandhi Road New Delhi 110 002, India

The discovery of antibiotics in the early part of 20th century and their spectacular success in combating infections and deaths created a complacency during the 1960s and 1970s. Over the past six decades, these ‘wonder drugs’ have played a critical role in reducing the global burden of communicable diseases. This success was however, overshadowed by the rapid backlash by the microbes resulting in a “relentless and dizzying rise of antimicrobial resistance”^1^. We seem to have come a full circle from the pre-antibiotic phase through a stage of euphoria to a seemingly frightening era of patients infected with multidrug resistant bacteria desperate for the elusive magic bullet^2^. In the recent past, especially, the emergence and spread of resistance in several microorganisms have rendered the management of many infectious diseases difficult.

## The problem

The wide and indiscriminate use of common anti-infective drugs has contributed substantially to the persistence of infections, as a major cause of morbidity and mortality. Multidrug resistant bacteria especially have emerged as a major health problem all over the world^3^,^4^. Resistance poses a growing threat to the treatment and control of endemic, epidemic-prone as well as pandemic diseases. Resistance in microorganisms costs money, livelihood and lives and threatens to undermine the effectiveness of health delivery programmes even in developed countries^5^. However, developing countries are hit hard with increasing reports of development of resistance to drugs commonly used to treat most of the communicable diseases. The emerging threat of resistance in malaria, tuberculosis (TB) and human immunodeficiency virus (HIV) infection is a huge impediment in achieving the Millennium Development Goals (MDGs) by 2015.

## The genesis and factors responsible

Resistance in microorganisms is defined as their unresponsiveness to the standard doses of drugs. Once developed, resistance is usually irreversible or very slow to reverse. Resistance is a naturally occurring, continuous but slow phenomenon. However, irrational use of antimicrobial agents accelerates this process and selects their resistant sub-populations which soon become the dominating member of the species. In fact, the more the usage of antibiotics, greater will the pace of emergence and selection of resistant bacteria.

The irrational use comprises inappropriate prescriptions which may be because of absence of standard treatment guidelines or physicians not adhering to these. It is estimated that 50 per cent of prescriptions by qualified physicians are inappropriate^1^. The WHO defines appropriate use of antibiotics as ”the cost-effective use of antibiotics, which maximizes clinical therapeutic effect while minimizing both drug-related toxicity and the development of antibiotic resistance”^3^. Even when the prescriptions are appropriate the patient may not be able to procure the drugs because of high cost or poor access. In developing countries an estimated 50 per cent of the those who need antimicrobials cannot access due to cost^6^. Many a times these drugs are not taken by the patient as per the recommended regimen or may be self administered when not required^6^. While data are not available from developed countries, in industralized economies, of the 80-90 per cent of antibiotic consumption in the community, almost half is due to incorrect indications, often viral infections^7^. Inexpensive and easily accessible testing facilities are lacking both to distinguish between bacterial and viral infections and, if bacterial, type the responsible species and its resistance pattern compounds the problem. In poor countries, almost 50 per cent of the patients fail to adhere to recommended regimen for various reasons including cost. Moreover, huge quantities of antimicrobials are used in veterinary sector for therapeutic as well as non-therapeutic purposes^3^. In some countries, more than 50 per cent of the national consumption of antimicrobials is for promoting the growth of food animals^6^. All these factors have the potential to provide an environment that selects out resistant subpopulations.

Antimicrobial resistance (AMR) now is far from being a local problem. It has international ramifications. In modern era of travel and trade, resistant organisms rapidly cross the man-made boundaries^8^ through humans or food chain. Resistant *Salmonella* species have entered several countries through food^9^,^10^.

## Situation of antimicrobial resistance in the South-East Asia (SEA) Region

Systematic studies to understand the epidemiology of antimicrobial resistance have not been undertaken in the SEA Region. However, information and data available for selected diseases/organisms show it to be a burgeoning and often neglected problem. Resistance to first-line anti-TB drugs has become a concern for national TB control programmes^11^. The population weighted mean of multi-drug resistant tuberculosis (MDR-TB) in the Region is 208 per cent (1.9-3.6%) among new cases and 18.8 per cent (13.3-24.3%) among previously treated cases^11^. It is estimated that around 180, 000 cases of MDR-TB reside/occur annually in this Region with more than 80 per cent of these being in Bangladesh, India, Indonesia, Myanmar and Thailand^11^. The drugs needed to treat MDR-TB are over 100 times more expensive than the first-line drugs used to treat non-resistant forms^11^. In some countries, the high cost of such replacement drugs is prohibitive, with the result that some of the MDR-TB cases can no longer be treated^11^.

The generic antiretroviral (ART) drugs available in the Region are contributing greatly towards improving the survival rate of patients worldwide and in rendering HIV as a chronic but a manageable condition. Although the response to ART drugs is excellent when these are delivered at health facilities^12^, there are reports of the emergence of resistance^13^ that is a serious cause of concern.

There has been a substantial change in the antimicrobial susceptibility of *Neisseria gonorrhoeae*. Thirty years back, gonorrhoea used to respond effectively to penicillin. Now the resistance to penicillin and fluoroquinolones is widespread across the Region^14^.

Resistant malaria has already become a major issue for a population of 400 million living in areas that expose them to a high risk of contracting it. Artemisinin-based combination therapies (ACT) have recently been introduced in virtually all countries in which malaria is endemic^15^. However, surveillance data from the Thai Ministry of Public Health indicate that clinical failures of artemisinin-based therapies exist in the Thai-Cambodian border, whereas efficacy with artesunate-mefloquine along the western borders of Thailand remains high^16^.

Pentavalent antimonials (SbV) have been successfully used for treatment of kala-azar since the last six decades. Since the 1970s, however, their conventional dosages have failed to achieve the desired results with 60 per cent unresponsiveness being reported with the WHO regimen in Bihar (India). Pentamidine initially used as a second-line drug, acquired resistance (25%) even with prolonged dosage^17^. The newer oral drug, miltefosine is a potent antileishmanial drug with a longer half-life, a property likely to delay resistance^17^. The evolution of resistance to this drug will cause havoc to the regional efforts to combat this disease.

Cholera bacilli have acquired resistance to a number of antimicrobials. The resistance spectrum varies in different locales. In areas around New Delhi (India) extensive resistance to furazolidone, co-trimoxazole and nalidixic acid has been noted^18^ while tetracycline has remained effective. On the other hand, in Bangladesh, tetracycline resistance has been found to be frequent in prevalent *Vibrio cholera*.^19^

*Streptococcus pneumoniae* is the most common causative agent of pneumonias in children and adults in Asia^20^. Till the1980s, almost all isolates of this organism used to be susceptible to penicillin. In 2006, in a hospital in Thailand, almost 69 per cent isolates of this bacterium were found to be penicillin resistant^21^.

Typhoid and paratyphoid fever continue to be important causes of illness and death, particularly among children and adolescents in the SEA Region where this disease is associated with poor sanitation and unsafe food and water. Shortly after the emergence of multidrug-resistant *S*. Typhi in this Region, case fatality rates approaching 10 per cent (close to 12.8% recorded in pre-antibiotic era) were reported^22^.

More than 50 per cent isolates of *Staphylococcus aureus* in hospital settings are now methicillin resistant. In a study undertaken in a 1000 bedded hospital in Thailand, 48 per cent patients with bacteraemia due to resistant *S. aureus* died^23^. Methicillin-resistant *S. aureus* (MRSA) is a major problem in hospital-associated infections in almost all countries in the SEA Region^24^.

Multiresistant klebsiellae, *Pseudomonas* and *Acinetobacter* species have given new dimensions to the problem of hospital-associated infections. *A. baumannii* has become an important pathogen in intensive care units. In a study done in Thailand, mortality in admitted patients due to imipenem-resistant *A. baumannii* was 52 per cent as compared to 19 per cent in those who were infected with the sensitive variant^25^. Presence of a drug resistant gene *bla*_NDM-1_ in several members of the family *Enterbacteriaceae* has given rise to organisms that are resistant to a large number of commonly used antimicrobial agents^26^.

Several salmonellae were isolated from chicken carcasses imported into Bhutan, 40 of 42 *Salmonella enteritidis* exhibited resistance to more than 2 drugs^9^. From clinically healthy cows in Thailand, 68 per cent of isolates of *S. enterica* were resistant to at least one antimicrobial and 6 per cent were multiresistant^27^. A spread of multiresistant *S. schwarzengrund* from chickens to humans in Thailand and from imported Thai food products to persons in Denmark and the United States has been well documented^10^.

## Consequences of AMR

AMR has several and severe consequences. The patient remains sick for a longer period thus requiring prolonged treatment usually with expensive and at times toxic drugs. Not only there is greater morbidity and mortality but the burden on health system also increases. The impact of modern technological and complex surgeries gets negated when the patient after successful intervention gets infected with resistant microorganisms. From the public health perspective, the patient acts as a reservoir of infection for a longer period thus putting at risk more members of community and health care workers. All these have substantial effect on economy at individual and societal levels. In fact, it is difficult to imagine effective newer surgical procedures, transplantations, prolonged chemotherapy for various cancers, care of critically ill young and the old, or prolonged treatment of the HIV-infected in the absence of measures towards effective containment of AMR.

## The need for new antibiotics

The need for new antibiotics to address the emerging resistance microorganisms cannot be overstated. A recent analysis by the European Centre for Diseases Prevention and Control and European Medicine Agency suggests of a near-empty antibiotic pipeline^28^. Only one or two drugs are under development that too in very early stages^5^. This is primarily because the industry is reluctant to invest in new antimicrobial drug development. There are various reasons for this some of which include^5^ *(i)* use of equally effective generic antibiotics for most infections as first line of treatment and use of new antibiotics generally as the last resort; *(ii)* antibiotics are used only for short duration as they are primarily used for curative purpose as compared to the drugs for chronic conditions used over long periods, even for a life time; *(iii)* emergence of rapid drug resistance rendering the antibiotic ineffective and hence less life span and lower returns on investment; *(iv)* difficulty in recruiting and conducting clinical trials due to several gray areas; and *(v)* relatively more regulatory hurdles for clinical trials. In fact all the global policies have consistently advocated the containment of resistance, rational and restricted use of antibiotics which are not industry-friendly.

There is some light at the end of the tunnel with some significant new initiatives. Some antimicrobial agents are under development and awaiting approval of the Food and Drug Administration, USA^2^. The European Commission has sought proposals for new antibiotic R&D for multidrug resistant Gram negative pathogens that was quickly followed with a joint EU-US Transatlantic Taskforce on Antimicrobial Resistance. Many other incentives to the industry to encourage antibiotic development are under various stages of global discussions^29^. As the overall returns of investments on antibiotics is never likely to touch a typical blockbuster drug for the large manufacturers, a model that could be considered is to engage small and medium pharmaceutical companies and develop antibiotics on a product development partnership models somewhat akin to those suggested for the development of drugs for neglected diseases. Public funding for new antibiotic R&D even in the rich countries has been negligible until recently. The call by the Infectious Diseases Society of America for a 10x20 initiative *viz*., development of 10 new antibiotics by 2020^30^ should trigger new R&D by the pharma companies.

## The future

We should recognize that the emergence of antimicrobial resistance is an inevitable consequence of the use of these life saving agents. And that the tools available are remarkably few and the pipeline for new products is near dry and it will be some time before new antimicrobials will become available. The emergence of the superbugs as the NDM 1 once again underscores the need for vigilance at community, local and national levels for the constant monitoring of AMR. There is a clear need for concerted action from all concerned in academia, hospitals and other health care settings, industry and the governments to work together in combating the emerging AMR, a cross cutting problem. Action is required on better diagnostics, educating the care givers and patients of the need for rational prudent use of antibiotics, work towards standard treatment protocols, dosage regimens and treatment durations. The problem of resistance is complex and encompasses biological, behavioural, technical, economic, regulatory and educational dimensions that require a comprehensive response.

A critical issue at the Regional level is the need for and difficulty in taking effective measures as the responsibility for health remains essentially a national problem. Accordingly, it requires ownership and active participation by several stakeholders, and a strategic approach with objectives that include establishment of a national alliance for prevention and control of antimicrobial resistance; institution of a surveillance system that captures the emergence of resistance, as well as the trends of its spread; and the utilization of antimicrobial agents in different settings; promotion of rational use of antimicrobial agents at all levels of health care and veterinary settings; strengthening infection control measures to reduce the disease burden; and supporting basic and operational research. The SEARO/WHO has recently developed one such strategy^31^.

Four areas which will need attention of national authorities pertain to governance, regulatory mechanisms, building national capacity in this area and mobilizing active participation of communities. A governance mechanism comprises establishment of a national alliance against AMR, designation of national focal point and establishment of a multi-sectoral National Steering Committee to guide national efforts.

The development and application of standard treatment guidelines in health and veterinary sectors, banning non-therapeutic use of drugs in animals and imposing restrictions on over-the-counter sale of antimicrobial agents are major activities for national regulatory agencies. Building core national capacity for monitoring antimicrobial use and resistance through national surveillance networks, rational use of antimicrobials by the prescribers, in reducing disease burden and undertaking appropriate operational research is critical. Finally educating communities for promoting adherence to recommended regimen and discouraging self prescription are equally important steps in mankind’s fight against AMR. At the country level, there is a need to integrate infection surveillance, prevention and control strategies and regulate and promote rational use of antibiotics. International agencies are also gearing up to combat this problem but some co-ordination towards orchestration of these global efforts could help. Recognizing the emerging importance of this subject and to enhance its visibility for an early action, “Antimicrobial Resistance” shall be the theme of World Health Day 2011. Far too long, antimicrobial resistance has been an unrecognized and neglected problem. The time to act is now.
